# Household Air Pollution from Solid Fuel Use and Risk of Adverse Pregnancy Outcomes: A Systematic Review and Meta-Analysis of the Empirical Evidence

**DOI:** 10.1371/journal.pone.0113920

**Published:** 2014-12-02

**Authors:** Adeladza K. Amegah, Reginald Quansah, Jouni J. K. Jaakkola

**Affiliations:** 1 Center for Environmental and Respiratory Health Research, Faculty of Medicine, University of Oulu, Oulu, Finland; 2 Public Health, Institute of Health Sciences, University of Oulu, Oulu, Finland; 3 Medical Research Center Oulu, University of Oulu and Oulu University Hospital, Oulu, Finland; 4 Respiratory Medicine Unit, Oulu University Hospital, Oulu, Finland; 5 Public Health, Department of Biomedical and Forensic Sciences, University of Cape Coast, Cape Coast, Ghana; 6 School of Public Health, University of Ghana, Legon, Ghana; Hasselt University, Belgium

## Abstract

**Background:**

About 41% of households globally, mainly in developing countries rely on solid fuels for cooking with consequences for fetal growth and development. Previous reviews were limited in scope, assessing only two outcomes (birth weight, stillbirth). With important evidence accumulating, there is a need to improve the previous estimates and assess additional outcomes. We conducted a systematic review and meta-analysis to evaluate the quality and strength of available evidence on household air pollution (HAP) and the whole range of adverse pregnancy outcomes.

**Methods:**

PubMed, Ovid Medline, Scopus and CINAHL were searched from their inception to the end of April 2013. All epidemiological study designs were eligible for inclusion in the review. The random-effects model was applied in computing the summary-effect estimates (EE) and their corresponding 95% confidence interval (CI).

**Results:**

Of 1505 studies screened, 19 studies satisfied the inclusion criteria. Household combustion of solid fuels resulted in an 86.43 g (95% CI: 55.49, 117.37) reduction in birth weight, and a 35% (EE = 1.35, 95% CI: 1.23, 1.48) and 29% (EE = 1.29, 95% CI: 1.18, 1.41) increased risk of LBW and stillbirth respectively.

**Conclusion:**

Combustion of solid fuels at home increases the risk of a wide range of adverse pregnancy outcomes. Access to clean household energy solutions is the surest way to combat HAP and mitigate their adverse effects.

## Introduction

Globally, 41% of households, mainly in developing countries in Asia and sub-Saharan Africa rely on solid fuels (coal and biomass) as their primary cooking fuel [1]. Combustion of solid fuels in simple household cookstoves emits considerably large amounts of health-damaging airborne pollutants including particulate matter (PM), carbon monoxide (CO) and polycyclic aromatic hydrocarbons (PAHs) [2] with poor ventilation of households often exacerbating the problem. Household air pollution (HAP) from solid fuel use was attributed to 4.5 million deaths globally in 2012, almost all in low and middle income countries [3]. There is also strong evidence linking household solid fuel use with acute respiratory infections in children, chronic obstructive pulmonary disease and lung cancer later in life [4]–[7]. Epidemiologic evidence linking ambient air pollution exposure with adverse pregnancy outcomes has been accumulating worldwide over the last two decades with several studies [8]–[12] also attempting to summarize the available evidence. There is however very limited evidence linking HAP exposure from solid fuel combustion with adverse pregnancy outcomes in spite of the widespread projection of HAP as the most important environmental exposure for pregnant women in developing countries.

The subject matter was reviewed previously by Pope et al. [13] and Misra et al. [14] albeit limiting the scope to only two outcomes; birth weight and stillbirth. Both studies provided evidence of an increased risk of low birth weight (LBW) and stillbirth with solid fuel use. Pope et al. [13] however pointed to limitations in the extent and quality of available evidence at the time of their study. With regards to Misra et al. [14] work, in spite of it being quite recent, it consisted of the very same studies previously reviewed by Pope and co-workers. A recent review proposing intervention estimates for child survival outcomes linked to HAP [15] attempted to update the estimates of Pope and co-workers and reported only one new eligible study on LBW with no new evidence on stillbirth found. A significant number of important new evidence has accumulated since Pope et al. [13] review which Bruce et al. [15] did not capture in their revised estimate. In such a rapidly evolving area these developments call for improving the previous estimates and assessing additional pregnancy endpoints. Also timely evaluation of methods and results of existing studies should help inform and improve the design of future studies. We therefore conducted a systematic review and meta-analysis of all studies examining the relations between solid fuel use at home and pregnancy outcomes to evaluate the quality and strength of the available evidence, and to identify gaps in knowledge and propose future research priorities.

## Methods

We conducted and report the study in accordance with the PRISMA (Preferred Reporting Items for Systematic Reviews and Meta-Analyses) guidelines [16].

### Search Strategy and Selection Criteria

We searched PubMed, Ovid Medline, Scopus and CINAHL from their inception to the end of April, 2013 with no language restrictions imposed. Medical Subject Heading (MeSH) terms and free text words were used to identify relevant studies from the databases. The search words applied are provided in Table 1. The search process combined the exposure and outcome terms systematically. Two independent investigators (AKA and RQ) initially screened the articles for eligibility based on the title and abstract. Articles were considered for inclusion if they were (a) original studies, (b) conducted in a human population and (c) investigated the relation between any of the exposures and outcomes listed in Table 1. Selected articles were retrieved in full and further assessed for eligibility. Studies were included if they either, (a) reported mean estimates for birth measurements among exposed and unexposed groups or mean differences between the two groups, or (b) reported effect estimates for the relation between an exposure and an outcome, or proportion of cases of any outcome among exposed and unexposed groups. We also reviewed the reference list of all included studies, and the three previous reviews [13]–[15] to identify additional eligible studies.

**Table 1:** Search words.

Exposure		Outcomes
MeSH terms	Free text words	MeSH terms
“indoor air pollution”	“household air pollution”	“pregnancy outcome”
biofuels	“household fuel”	“birth weight”
biomass	“domestic fuel”	“low birth weight”
coal	“cooking fuel”	“premature birth”
wood	“cooking smoke”	“premature infant”
charcoal	“solid fuel”	“fetal growth retardation”
cooking	firewood	“fetal development”
	“crop residue”	“gestational age”
	“biomass fuel”	“small for gestational age”
	“biomass smoke”	“fetal mortality”
	“wood fuel”	“fetal death”
	“wood smoke”	“perinatal mortality”
	“charcoal smoke”	stillbirth
		“embryo loss”
		“spontaneous abortion”
		“congenital abnormalities”
		“neural tube defects”

### Data Extraction and Quality Assessment of Studies

Data from eligible studies were extracted independently by two investigators (AKA and RQ) onto a form. Disagreements during synthesis of the data extracted were resolved through discussion with the third investigator (JJKJ) serving as an adjudicator. We contacted authors for clarifications where needed. Methodological quality of the included studies was assessed by using the original Newcastle-Ottawa scale (NOS, maximum of 9 stars) for case control and cohort designs, and an adapted NOS (maximum of 6 stars) for cross-sectional designs. In evaluating the adequacy of confounding control in the included studies, we put together a shortlist of core confounders that needed to be adjusted for in the analysis. The shortlist included maternal age and obstetric history, maternal nutrition and anthropometry, socioeconomic status, and active and passive smoking.

### Statistical Analysis

We anticipated heterogeneity between the studies due to differences in study design, and geographical settings and populations studied. We therefore applied the random-effects model which accounts for both within and between study heterogeneity in computing the summary-effect estimates. With regards to studies providing multiple effect estimates (e.g. wood, coal and other solid fuels), we first combined the effect estimates using fixed-effects model and applied the single effect estimate in the overall meta-analysis. We quantified heterogeneity using the Cochran Q (X^2^) test and the *I^2^* statistic with a value>50% deemed to indicate substantial heterogeneity. Forest plots were also visually assessed. We explored possible sources of heterogeneity by conducting subgroup analysis and meta-regression. We conducted sensitivity analysis by limiting the analysis to high quality studies; 7 or more stars on the original NOS for case-control and cohort studies, and 6 stars on the adapted NOS for cross-sectional studies. Publication bias was investigated by visually inspecting funnel plots for asymmetry, and applying the Begg's and Egger's tests. We accounted for publication bias using the trim and fill method. Analyses were conducted using Stata version 9.0 (Stata Corporation, College Station, TX, USA).

## Results

**Figure 1:**
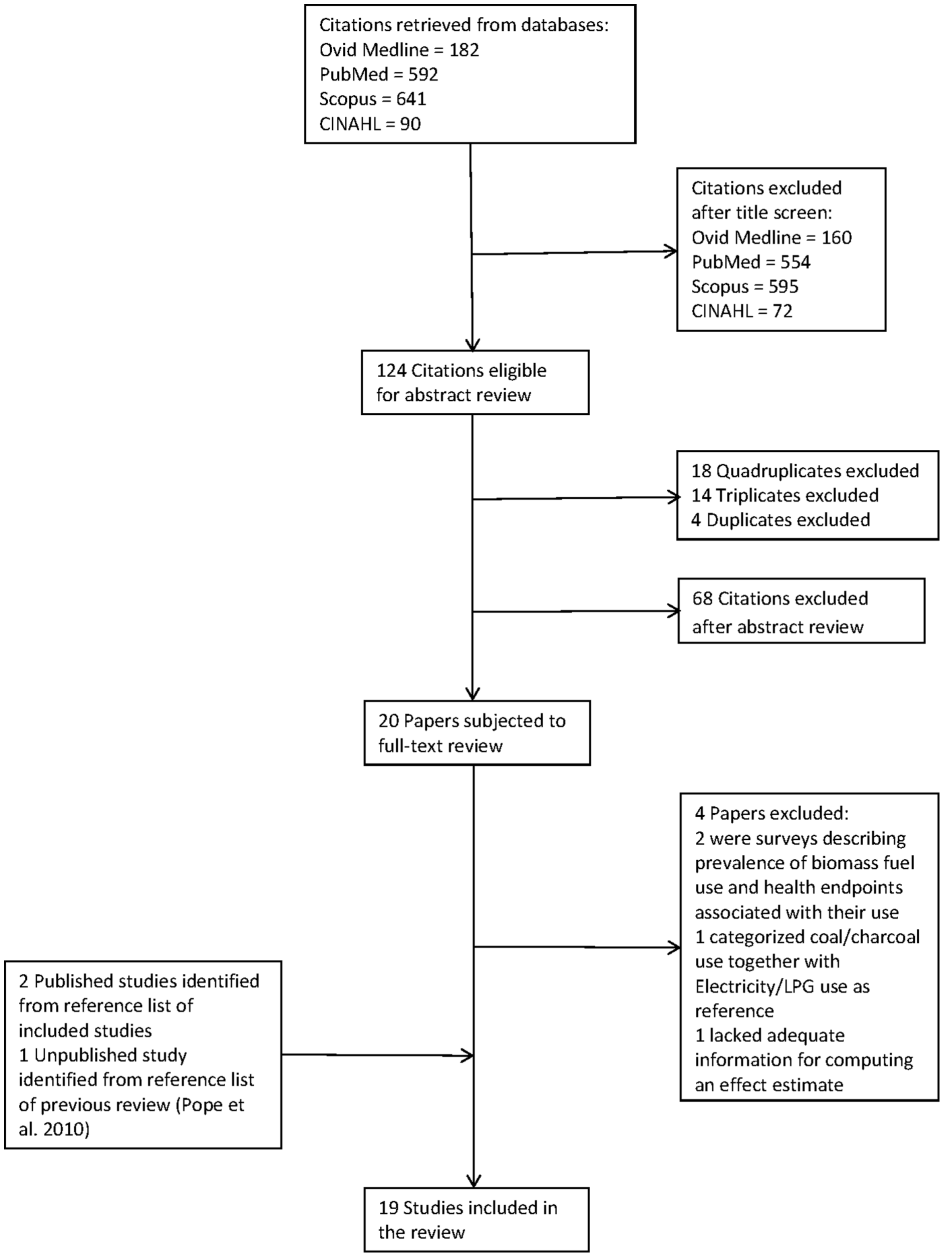
Flowchart of search strategy and selection of studies for inclusion in review.

A flowchart of the study selection process is depicted in Figure 1. A total of 19 studies were included in the review.

### Characteristics of Included Studies

The characteristics of included studies are presented in Table 2. Seven studies employed a cross-sectional design, of which five analyzed nationwide demographic and health survey (DHS) data. Cohort design was applied by five studies, of which four were undertaken prospectively. One study was a randomized controlled trial (RCT). Six studies were case-control studies of which three adopted a matched design. Ten studies were conducted in South Asia mostly in India with only two studies conducted in sub-Saharan Africa. Ten of the included studies were published after the year 2010.

**Table 2:** Characteristics of included studies.

First author, Year (Reference No.)	Location	Setting	Design	Exposure assessment	Outcome	Adequacy of confounding control	Effect estimate	Quality score
Boy, 2002 (24)	Quetzaltenango Province, Western Guatemala	Rural and urban	Cross-sectional	Interview. Type of cooking fuels and combustion method applied (open fire or chimney stove) if wood or coal was used	BW, LBW	Yes	Adjusted mean difference: 63 g (95% CI: 0.4, 126) Unadjusted OR^a^: 1.21 (95% CI: 0.88, 1.66)	6/6
Mishra, 2004 (31)	Zimbabwe	Nationwide	Cross-sectional	Interview. Main household cooking fuels	BW, LBW	Yes	Adjusted β: All births 175 g (95% CI: −300, −50), Health card holders 120 g (95% CI: −301, 61), Maternal recall 183 g (95% CI: −376, 10) Unadjusted OR^a^: 1.12 (95% CI: 0.81, 1.54)	5/6
Mishra, 2005 (21)	India	Nationwide	Cross-sectional	Interview. Main and other types of household cooking fuels, and fuel mixing ascertained.	Stillbirth	No	Adjusted OR: Biomass fuel 1.44 (95% CI: 1.05, 1.97), Fuel mix 1.19 (*p*>0.05)	4/6
Siddiqui, 2005 (32)	Sindh province, Southern Pakistan	Rural and urban	Prospective cohort	Interview. Main cooking fuel used (wood, NG).	LBW, Stillbirth, Miscarriage	No	Adjusted OR: LBW 1.77 (95% CI: 1.20, 2.50), Stillbirth 1.90 (95% CI: 1.10, 3.20)	7/9
Siddiqui, 2008 (25)	Rehri Goth, Pakistan	Semi-rural	Retrospective cohort	Interview. Type of cooking fuel (wood, NG) at time of interview, during index pregnancy and any changes since the pregnancy. Cooking practices and kitchen ventilation ascertained.	BW, LBW	Yes	Adjusted OR: 1.86 (95% CI: 1.11, 3.14) Adjusted β: 82 g (−170, 9)	8/9
Tielsch, 2009 (22)	Tamil Nadu, South India	Rural	Prospective cohort	Interview. Type of cooking fuel	BW, LBW, PTB, SGA, Stillbirth	Yes	Adjusted mean difference 104.5 g (95% CI: −140.1, −68.9) Adjusted RR: LBW 1.49 (95% CI: 1.25, 1.77), PTB 1.43 (95% CI: 1.11, 1.84), SGA 1.21 (95% CI: 1.11, 1.31), Stillbirth 1.34 (95% CI: 0.76, 2.36)	8/9
Yucra, 2011 (23)	Abancay and Huancavelica Districts, Peru	Urban	Matched case-control	Interview. Type of cooking fuel, fuel mixing, cooking practices, and kitchen ventilation ascertained	LBW, PTB, Adverse perinatal outcome (LBW and PTB combined)	Yes	Adjusted OR (Biofuel): LBW 3.73 (95% CI: 1.14, 12.1), PTB 1.59 (95% CI: 0.41, 6.18), Adverse perinatal outcome 2.54 (95% CI: 1.06, 6.11) Adjusted OR (Gas + Biofuel): LBW 1.66 (95% CI: 0.45, 5.99), Adverse perinatal outcome 1.40 (95% CI: 0.54, 3.60)	5/9
Thompson, 2011 (17)	Guatemala	Rural	Randomized controlled trial	48-hour CO levels measured. Actual stove type (chimney stove, open fire) used in pregnancy ascertained.	BW, LBW	No	Adjusted β: 89 g (95% CI: −27, 204) Adjusted OR: 0.74 (95% CI: 0.33, 1.66)	7/9
Sreeramareddy, 2011 (19)	India	Nationwide	Cross-sectional	Interview. Main household cooking fuels	BW, LBW	Yes	Adjusted OR: 1.21 (95% CI: 1.06, 1.32) Adjusted mean difference 39.9 g (SD 13.4)	5/6
Stankovic, 2011a (29)	Nis and Niska Banja, Serbia	Urban	Prospective cohort	Interview. Exposure to smoke from heating fuels (wood, coal)	BW, Birth length	No confounding control but excluded certain category of mothers	Unadjusted mean difference: BW 99.1 g (95% CI^a^: 4.1, 194.1), Birth length 0 cm (*p*> 0.05)	6/9
Stankovic, 2011b (34)	Nis and Niska Banja, Serbia	Urban	Prospective cohort	Interview. Exposure to smoke from heating fuels (wood, coal)	Premature labour, Miscarriage	No confounding control but excluded certain category of mothers	Unadjusted OR: Miscarriage 1.12 (95% CI: 0.70, 1.77), Premature labour 1.07 (95% CI: 0.76, 1.52)	6/9
Abusalah, 2012 (27)	Gaza Strip	Urban	Matched case-control	Interview. Exposure to wood fuel smoke from cooking	BW, LBW	Yes	Adjusted Matched OR: 2.3 (95% CI: 1.2, 4.7) Adjusted β: 186 g (95% CI: −354, −19)	6/9
Li, 2011 (26)	Shanxi Province, China	Rural	Population-based matched case-control	Interview. Primary cooking and heating fuels, cooking and heating practices, kitchen location, and housing ventilation ascertained.	Neural tube defect	Yes (in dose-response analysis). No confounding control in main analysis	Unadjusted OR: Cooking 1.9 (95% CI: 1.4, 2.6), Heating 1.7 (95% CI: 0.2, 19.1)	5/9
Epstein, 2013 (18)	India	Nationwide	Cross-sectional	Interview. Type of primary household fuel	BW, LBW	Yes	Adjusted OR: Biomass fuels 1.24 (95% CI: 1.04, 1.48), Kerosene 1.51 (95% CI: 1.08, 2.12), Coal 1.57 (95% CI: 1.03, 2.41), Solid fuels^b^ 1.28 (95% CI: 1.09, 1.51) Unadjusted mean difference: Biomass fuels 78.1 g (95% CI: −100.4, 55.7), Kerosene 106.5g (95% CI: −152.5, 59.4), Coal 110 g (95% CI: −188.5, 31.5), Solid fuels^b^ 88.78 (95% CI: −25.13, 152.43)	5/6
Lakshmi, 2013 (20)	India	Nationwide	Cross-sectional	Interview. Type of cooking and lighting fuel	Stillbirth	Yes	Adjusted PR: Kerosene 1.36 (95% CI: 1.10, 1.67), Wood 1.24 (95% CI: 1.08, 1.41), Other 1.23 (95% CI: 1.05, 1.44), Solid fuels^b^ 1.24 (95% CI: 1.12, 1.37)	4/6
Amegah, 2012 (28)	Accra, Ghana	Urban	Cross-sectional	Interview. Cooking fuel (charcoal, LPG) used during pregnancy, fuel mixing, cooking practices, kitchen ventilation, and garbage burning at home ascertained.	BW, LBW	Yes	Adjusted RR: Charcoal 1.41 (95% CI: 0.62, 3.23), Charcoal & LPG 1.09 (95% CI: 0.41, 2.93) Adjusted β: Charcoal 243 g (95% CI: −496, 11), Charcoal & LPG 109 g (95% CI: −406, 188)	5/6
Mavalankar, 1991 (33)	Ahmedabad, Western India	Urban	Case-control	Interview. Exposure to cooking smoke.	Stillbirths	Yes	Adjusted OR: 1.5 (95% CI: 1.0, 2.1)	5/9
Mavalankar, 1992 (30)	Ahmedabad, Western India	Urban	Case-control	Interview. Exposure to cooking smoke.	Term and preterm LBW	Yes	Unadjusted OR: Term LBW 1.23 (95% CI^a^: 1.01, 1.49), Preterm LBW 1.49 (95% CI^a^: 1.23, 1.81), All cases^a^ 1.35 (95% CI: 1.15, 1.58) Adjusted estimates not reported.	5/9
Samaraweera, 2010 (35)	Colombo, Sri Lanka	Urban	Case-control	Interview. Exposure to cooking smoke from firewood use in a kitchen without a chimney	Miscarriage (Trimester-specific)	No	Unadjusted OR: First trimester 2.10 (95% CI: 0.90, 4.87). Adjusted OR (Second trimester): 3.83 (95% CI: 1.50, 9.90) All cases^a^ 2.54 (95% CI: 1.39, 4.63)	5/9

BW: birth weight; CI: confidence interval; CO: carbon monoxide; LBW: low birth weight; LPG: liquefied petroleum gas; OR: odds ratio; PR: prevalence ratio; PTB: preterm birth; RR: risk ratio; SD: standard deviation; SGA: small for gestational age.

a Computed from data reported in manuscript.

b Combined estimate for the individual effect estimates reported using fixed-effects model.

With the exception of the RCT [17] exposure to HAP was assessed indirectly through the use of questionnaires to solicit information on primary cooking and heating fuels used by maternal households routinely or during the index pregnancy. Thompson et al. [17] attempted to measure CO levels in households but obtained very few measurements and as a result relied on actual stove type (chimney stove vs. open fire) used. Six studies collected information on the use of one specific solid fuel type with wood fuel being the most studied. Ten studies collected information on two or more solid fuels with all the studies grouping the individual fuels together in the assessment of HAP exposure. Six studies collected information on kerosene use and categorized this fuel either as high pollution [18]–[20] or low pollution [21]–[23]. Epstein et al. [18] and Lakshmi et al. [20] did however assess their independent effects. Six studies [23]–[28] collected other exposure data such as cooking habits and practices, and ventilation of cooking area in an attempt to properly characterize exposures. Three studies [21], [23], [28] collected information on use of combination of biomass and cleaner fuels.

Birth weight was ascertained by 14 studies and was measured at home in four studies that were community-based [17], [22], [24], [25] within 48–72 hours using infant scales. Five studies [23], [27]–[30] were hospital based and obtained birth weight measures from hospital records. The studies that analyzed DHS data [18], [19], [31] obtained birth weight measures from health cards or mother's recall in situations where health card was unavailable. Stillbirth was ascertained by five studies [20]–[22], [32], [33] with all except the unpublished study [32] conducted in India. Three studies [22], [23], [34] investigated preterm birth (PTB, <37 weeks gestation) with gestational age estimated by the last menstrual period method in all the studies. Tielsch et al. [22] also studied SGA (newborns below the 10^th^ percentile of weight for gestational age at birth). Two studies [34], [35] measured miscarriage with one study [26] ascertaining neural tube defect. Two studies [28], [30] assessed term LBW; a proxy for intrauterine growth retardation (IUGR).

### Methodological Quality of Included Studies

Selection bias was generally minimized in all the included studies as the studies were largely representative of their source population and reported high response rates. Information bias was a potential problem in all the studies due to the reliance on interview methods in assessing HAP exposure. The prospective cohort studies collected exposure data at baseline but it is unclear whether fuel choices of mothers remained relatively stable throughout pregnancy. The retrospective cohort study [25], in contrast, ascertained changes in fuel type and cooking frequency/duration during pregnancy and restricted the analysis to women who consistently used the same fuel type during the index pregnancy. There is however the potential for information bias due to the retrospective data collection. The DHS surveys collected information on primary cooking fuel of households which certainly raises doubt as to whether same fuel was used by mothers during the index pregnancy. Of the case-control studies, only one study [27] blinded interviewers to case/control status in the ascertainment of exposure.

Outcomes were objectively measured at home/hospital or ascertained from hospital records for majority of the included studies. There is a strong potential for outcome measurement bias in two studies [19], [31] which relied on maternal recall of child size at birth to respectively estimate birth weight of 47% and 84% of infants included in the ratio scale birth weight analysis due to unavailability of health cards. Sreeramareddy et al. [19] further relied on maternal judgment of baby size at birth in classifying LBW babies due to lack of birth weight data on almost 60% of the study infants with a strong likelihood of outcome misclassification. Epstein et al. [18] however included only newborns that had birth weights recorded on health cards thereby excluding newborns who were not delivered at health facilities, an approach that has obvious implications for sample representativeness and generalizability of study findings. Of the studies that ascertained stillbirth, only one study [21] provided a case definition in their report. Of the two studies ascertaining miscarriage, Samaraweera and Abeysena [35] provided a case definition whereas Stankovic et al. [34] did not. The time of measurement of birth weight of newborns delivered at home in the community-based studies [17], [22], [24], [25] raise doubts about their acceptability as actual birth weight of these infants. Boy et al. [24] however excluded more remote communities to ensure neonates were examined within 72 hours and has obvious implications for sample representativeness and generalizability of study findings. Tielsch et al. [22] and Thompson et al. [17] on the other hand respectively excluded neonates not weighed within 72 hours (18.1%) and 48 hours (31.5%) from the analysis which are also likely to bias the effect estimates.

All but two studies [29], [34] adjusted for a range of potential confounders in the analysis including demographic, household and socioeconomic factors; maternal nutritional, health and lifestyle factors; neonatal characteristics; and second-hand smoke exposure. Stankovic and colleagues [29], [34] however included in their studies only mothers who were non-smokers and reported no occupational air pollution exposure, as well as excluding mothers with previous underlying diseases (hypertension, diabetes, anemia etc.) and other present pathological problems (infections, cervical insufficiency etc.). The studies that analyzed DHS data and three other studies [22], [30], [33] were characterized by adjustment for several covariates but their large sample sizes means loss of statistical efficiency might not be a concern. Based on our *a priori* criteria, of the included studies that adjusted for potential confounders, confounding control was considered inadequate in four studies [17], [21], [32], [35].

Overall, applying the NOS scale, two studies [22], [25] were rated as very high quality (case-control/cohort - 8 or more stars), three studies [17], [24], [32] as high quality (case-control/cohort -7 stars; cross-sectional - 6 stars), twelve studies [18], [19], [23], [26]–[29], [30], [31], [33]–[35] as satisfactory quality (case-control/cohort - 5 or 6 stars; cross-sectional - 5 stars) and two studies [20], [21] as low quality (<5 stars for both case-control/cohort and cross-sectional).

### Summary-Effect Estimates, Evidence of Statistical Heterogeneity and Publication Bias

**Figure 2:**
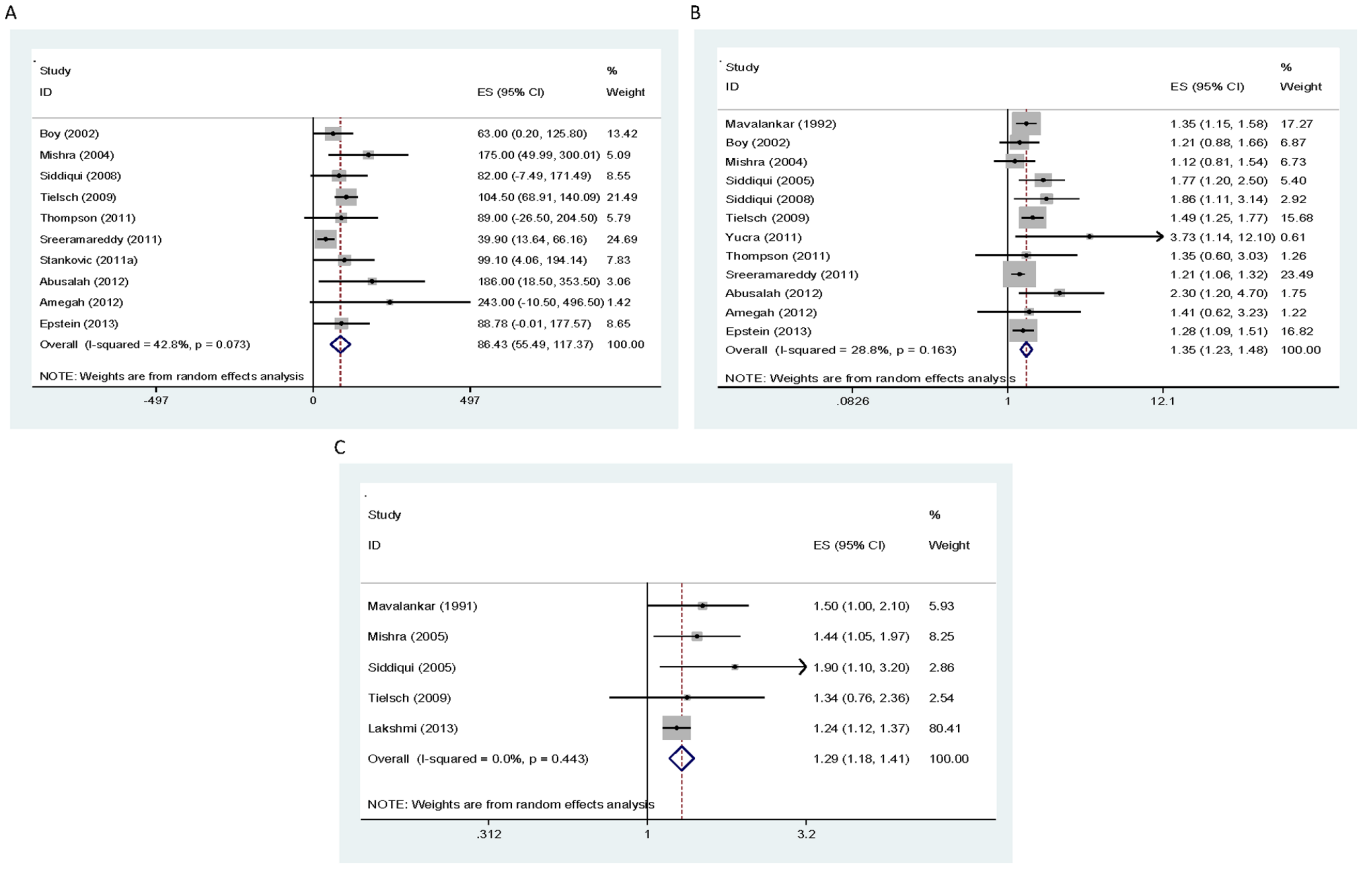
Forest plot showing the effect of household solid fuel use on birth weight (A), low birth weight (B) and Stillbirth (C). ES: effect size; CI: confidence interval.

**Figure 3:**
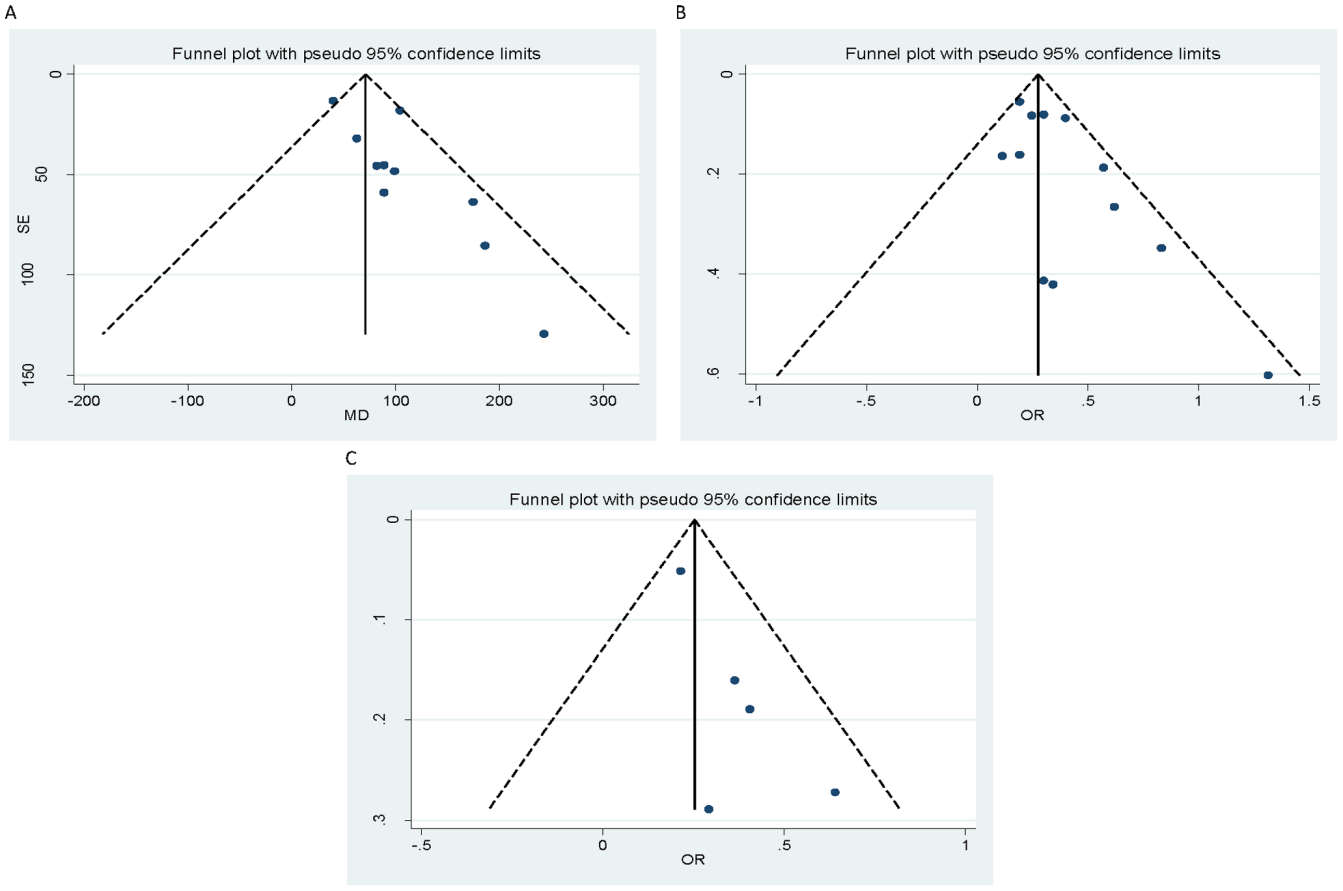
Funnel plot for the relation between household solid fuel use and birth weight (A), low birth weight (B) and stillbirth (C).

On the relation of solid fuel use with average birth weight, the study conducted in Ghana [28] reported the highest effect size (243 g) with an Indian study [19] reporting the lowest effect size (39.9 g). On the relation of solid fuel use with LBW, the study conducted in Peru [23] reported the highest and most extreme estimate (OR = 3.73, 95% CI: 1.14, 12.1). The summary-effect estimate was respectively −86.43 g (95% CI: −117.37, −55.49) and 1.35 (95% CI: 1.23, 1.48) for birth weight and LBW. We observed evidence of low statistical heterogeneity in both analyses (Table 3, Figure 2). We also found evidence of publication bias in both analyses (Figure 3) with both the Begg's and Egger's tests (Table 4) confirming the funnel plot asymmetry observed. The adjusted estimates were attenuated (Table 4). Both sensitivity analyses resulted in an increase in the summary-effect estimate with no evidence of heterogeneity observed (EE = −92.84, 95% CI: −121.20, −64.47, *I^2^* = 0.0% and EE = 1.49, 95% CI: 1.30, 1.70, *I^2^* = 0.0% respectively).

**Table 3:** Summary-effect estimates (EE) for the relation of solid fuel use with adverse pregnancy outcomes.

		Random-effects model		Heterogeneity		
Outcome	No. of studies	EE	95% CI	Cochran X^2^	p value	*I^2^* (%)
Birth weight	10	−86.43	−117.37, −55.49	15.73	0.073	42.8
LBW	12	1.35	1.23, 1.48	15.46	0.163	28.8
Stillbirth	5	1.29	1.18, 1.41	3.73	0.443	0.0
PTB	3	1.30	1.06, 1.59	1.84	0.398	0.0
IUGR	2	1.23	1.01, 1.49	0.02	0.892	0.0
Miscarriage	2	1.65	0.74, 3.67	4.46	0.035	77.6

CI: confidence interval; EE: summary-effect estimates; IUGR: intrauterine growth retardation; LBW: low birth weight; PTB: preterm birth.

Birth weight estimate is in grams.

**Table 4:** Test for publication bias and adjusted summary-effect estimates.

	Begg's test		Egger's test			Random-effects model		
Outcome	z	p value	Bias coefficient	95% CI	p value	No. of studies	EE	95% CI
Birth weight	1.52	0.128	1.471	0.196, 2.746	0.029	16	−53.95	−86.96, −20.94
LBW	1.78	0.075	1.189	0.810, 2.297	0.038	16	1.29	1.16, 1.44
Stillbirth	0.98	0.327	1.217	−0.039, 2.473	0.054	8	1.25	1.11, 1.40

CI: confidence interval; EE: summary-effect estimates; LBW: low birth weight.

Birth weight estimate is in grams.

Regarding the other outcomes investigated, with the exception of miscarriage, we observed no evidence of statistical heterogeneity in the analysis (Table 3). For PTB, IUGR and stillbirth, the summary-effect estimate was 1.30 (95% CI: 1.06, 1.59), 1.23 (95% CI: 1.01, 1.49) and 1.29 (95% CI: 1.18, 1.41) respectively. The summary-effect estimate for miscarriage (EE = 1.65, 95% CI: 0.74, 3.67) was substantially elevated but not statistically significant. The small number of studies ascertaining PTB, IUGR and miscarriage made an investigation of publication bias impractical. Evidence of publication bias was noted in the stillbirth analysis (Figure 3) with the adjusted estimate also attenuated (Table 4). Only one study (26) investigated neural tube defect and reported an unadjusted OR of 1.9 (95% CI: 1.4, 2.6) and 1.7 (95% CI: 0.2, 19.1) for cooking and heating with coal respectively.

### Sources of Heterogeneity between Included Studies

Results of the sub-group analysis which was performed for only birth weight and LBW due to the small number of studies investigating the other outcomes are presented in Tables 5 and 6 respectively. For birth weight, the summary-effect estimates for the Latin American and South Asian studies were much lower than the estimates for the Sub-Saharan African studies. The opposite was noted for the LBW outcome. Whereas evidence of heterogeneity between the South Asian studies was observed for both outcomes, for the Latin American studies it was observed for only the LBW outcome. Regarding the study setting (rural vs. urban), for both outcomes, the combined estimate for studies conducted in urban areas was higher than the combined estimate for studies conducted in rural areas. Also for both outcomes, the summary-effect estimates computed for studies applying cohort design was higher than estimates summarized for studies applying cross-sectional design. Of the cross-sectional studies, and regarding the birth weight outcome, the summary estimate for the primary studies was higher than the estimate computed for the studies analyzing DHS data.

**Table 5:** Summary-effect estimate for the relation of solid fuel use with birth weight stratified according to the study characteristics.

		Random-effects model		Heterogeneity		
Study characteristic	No. of studies	EE	95% CI	Cochran X^2^	p value	*I^2^* (%)
Geographic location						
South Asia	4	−74.81	−116.97, −32.65	8.65	0.034	65.3
Latin America	2	−68.93	−124.10, −13.76	0.15	0.698	0.0
Sub-Saharan Africa	2	−188.30	−300.42, −76.19	0.22	0.637	0.0
Eastern Europe	1	−99.1	−194.1, −4.1			
Middle East	1	−186	−354, −19			
Study setting						
Rural	3	−100.49	−132.28, −68.69	0.25	0.882	0.0
Urban	3	−132.06	−210.65, −53.47	1.60	0.450	0.0
Study design						
RCT	1	−89	−204, 27			
Cohort	3	−101.18	−132.41, −69.94	0.21	0.900	0.0
Case control	1	−186	−354, −19			
Cross-sectional	5	−75.91	−122.93, −28.88	7.49	0.112	46.6
Primary study	2	−109.38	−263.68, 44.91	1.82	0.177	45.2
Secondary analysis	3	−81.22	−151.48, −10.96	5.14	0.077	61.1
Exposure assessment (Handling of solid fuel data)						
Grouped together	6	−79.72	−115.21, −44.24	11.75	0.038	57.4
Separated/Specific fuels studied	4	−114.96	−177.13, −52.79	2.20	0.532	0.0
Outcome ascertainment (Place of measurement)						
Hospital	5	−113.71	−169.68, −57.75	2.11	0.715	0.0
Home	3	−100.49	−132.28, −68.69	0.25	0.882	0.0
Quality score						
Very High	2	−101.43	−134.50, −68.35	0.21	0.647	0.0
High	2	−68.93	−124.10, −13.76	0.15	0.698	0.0
Satisfactory	6	−101.56	−159.64, −43.49	10.64	0.059	53.0

CI: confidence interval; EE: summary-effect estimates.

EE are in grams.

**Table 6:** Summary-effect estimate for the relation of solid fuel use with low birth weight stratified according to the study characteristics.

		Random-effects model		Heterogeneity		
Study characteristic	No. of studies	EE	95% CI	Cochran X^2^	p value	*I^2^* (%)
Geographic location						
South Asia	6	1.36	1.24, 1.50	8.62	0.125	42.0
Latin America	3	1.47	0.89, 2.44	3.26	0.196	38.7
Sub-Saharan Africa	2	1.15	0.86, 1.56	0.26	0.610	0.0
Middle East	1	2.30	1.20, 4.70			
Study setting						
Rural	3	1.52	1.29, 1.78	0.71	0.701	0.0
Urban	4	1.66	1.14, 2.41	4.87	0.182	38.4
Study design						
RCT	1	1.35	0.60, 3.03			
Cohort	3	1.56	1.34, 1.82	1.16	0.559	0.0
Case control	3	1.86	1.07, 3.22	4.87	0.088	58.9
Cross-sectional	5	1.22	1.13, 1.33	0.74	0.946	0.0
Primary study	2	1.23	0.92, 1.66	0.12	0.734	0.0
Secondary analysis	3	1.22	1.12, 1.34	0.63	0.731	0.0
Exposure assessment (Handling of solid fuel data)						
Grouped together	7	1.29	1.18, 1.41	8.43	0.208	28.8
Separated/Specific fuels studied	5	1.75	1.40, 2.18	1.19	0.880	0.0
Ascertainment of Outcome (Place of measurement)						
Hospital	5	1.39	1.18, 1.64	5.60	0.231	28.6
Home	3	1.52	1.29, 1.78	0.71	0.701	0.0
Quality score						
Very High	2	1.52	1.29, 1.80	0.63	0.428	0.0
High	3	1.42	1.10, 1.85	2.38	0.305	15.9
Satisfactory	7	1.29	1.16, 1.43	8.08	0.232	25.7

CI: confidence interval; EE: summary-effect estimates.

Regarding information collected on solid fuel types used in households, for both outcomes, the combined estimate recorded for studies that grouped the various fuels together was lower than the estimate summarized for studies that assessed one specific fuel or investigated the fuels independently. Evidence of heterogeneity between the studies that grouped the individual solid fuels was noted. For the LBW outcome, the summary-effect estimates increased as the quality of the studies increases (Table 6). An inconsistent trend was observed for the birth weight outcome (Table 5).

In the meta-regression models; study design (cross-sectional: β = −0.240, p = 0.022), exposure assessment (β = −0.342, p = 0.037) and study quality (β = −0.160, p = 0.086) were the covariates associated with heterogeneity observed in the LBW analysis. None of the covariates was statistically associated with the observed heterogeneity in the birth weight analysis.

## Discussion

Our results indicate that household use of solid fuels adversely affects pregnancy outcomes. Solid fuel use leads to an 86.43 g (95% CI: 55.49, 117.37) reduction in birth weight and a 35% (EE = 1.35, 95% CI: 1.23, 1.48) increased risk of LBW. We also found evidence of an increased risk of stillbirth, PTB, IUGR and miscarriage for use of solid fuel at home.

### Validity of Results

Our study is based on much more information (>50%) than the previous reviews and certainly has much higher statistical power. Some of the included studies however had major methodological drawbacks with obvious implications for the quality of the evidence reported. We therefore conducted sensitivity analysis by excluding studies rated as satisfactory and low quality to assess the robustness of our results. The summary-effect estimates from the sensitivity analyses were not markedly different from the overall estimates. We also conducted subgroup analysis and meta-regression to elaborate the observed heterogeneity in the analysis. This was to ensure the inherent differences among the individual studies that were likely to impact on the validity and usefulness of the summary estimates are not ignored. Of the covariates explored, study design, exposure assessment method applied, and the methodological quality appear to be the variables likely to impact on the interpretation of our findings. For these three covariates we observed very consistent results in the stratified analysis. The results obtained from the meta-regression corroborated the findings from the LBW stratified analysis.

**Figure 4:**
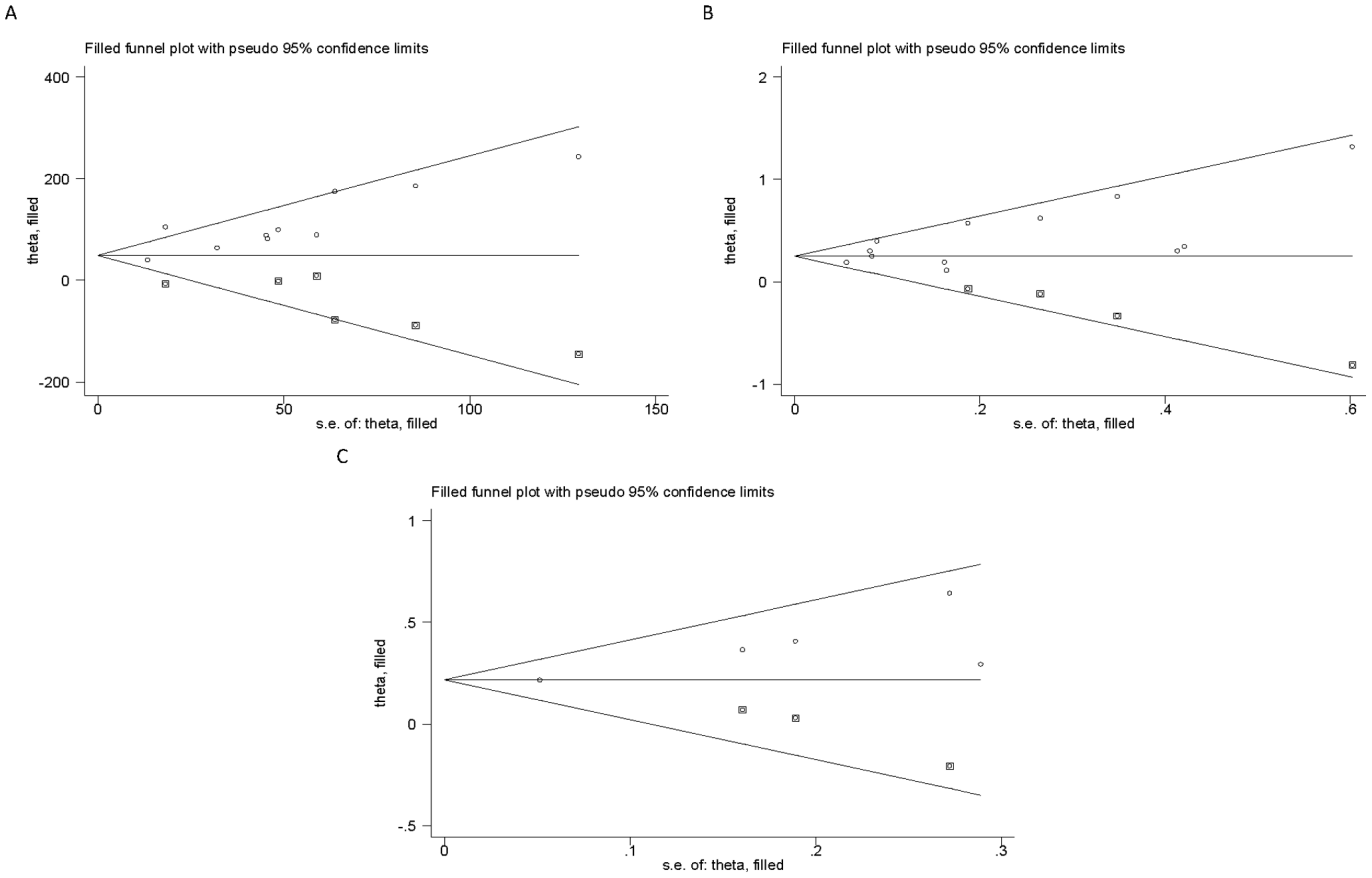
Filled funnel plot for the relation between household solid fuel use and birth weight (A), low birth weight (B) and stillbirth (C).

A major validity concern of meta-analysis is the tendency of overestimating the magnitude of the true effect size due to publication bias. We therefore investigated publication bias and accounted for the bias where evidence of its presence was found (Figure 4). With regards to the LBW and stillbirth outcomes, the adjusted estimates obtained after controlling for publication bias were quite similar to the crude estimates. Regarding the birth weight outcome, the adjusted estimate obtained was attenuated.

### Synthesis with Previous Knowledge

We found household combustion of solid fuels to be associated with an 86 g reduction in birth weight. Accounting for publication bias however reduced the effect size to 54 g suggesting a possible overestimation of the summary estimate in the meta-analysis. Pope et al. [13] previously reported a 96.6 g reduction in birth weight for HAP exposure from solid fuel use with no evidence of publication bias found. Our results however show that the effect estimate varies from one population to another and also along the rural-urban divide, possibly due to the exposure intensity which are dependent on the fuel choices. For instance, whereas charcoal use is widespread in sub-Saharan Africa especially in urban areas, wood which is more polluting is commonly used in South Asia. Rural African settings patronize wood and crop residues mostly. A multi-country study reported South Asian women to commonly use wood (49.1–89.7%), crop residue and animal dung as domestic fuel with African women using charcoal mostly (85.4–93.5%) [36]. Zulu and Richardson [37] also reported that more than 80% of urban households in sub-Saharan Africa use charcoal as their main cooking fuel. However, in the sub-group analysis, the summary estimate for studies conducted in sub-Saharan Africa was quite larger than the estimate obtained for the South Asian studies. Also the summary estimate computed for studies conducted in urban settings was higher than the estimate reported for studies conducted in rural settings. This finding could be attributed in part to the grouping together of the individual fuels in the assessment of HAP exposure and the inconsistent categorization of kerosene. Differences in birth weight distributions could also partially explain the heterogeneity of effect estimates, but it should be pointed out that an absolute effect of HAP exposure could indicate a more severe effect in a population with lower levels of birth weight. Our finding of 35% (EE = 1.35, 95% CI: 1.23, 1.48) increased risk of LBW for solid fuel use is consistent with the findings of Pope et al. [13] study and the revised estimate by Bruce et al. [15].

We also found household use of solid fuels to be associated with a 29% (EE = 1.29, 95% CI: 1.18, 1.41) increased risk of stillbirth and was quite lower than the effect estimate previously reported by Pope et al. [13]. In addition to the four studies reviewed by Pope and colleagues, our search strategy also identified one other recent study [20]. This study was the largest and also reported the smallest and most precise estimates (Combined Prevalence Ratio = 1.24, 95% CI: 1.12, 1.37) thereby gaining the bulk of the weight (80.41%) in the overall meta-analysis and pulling the summary estimate towards the null as a result. This study and two other studies [21], [33] were however excluded in the sensitivity analysis resulting in a much higher stillbirth risk of 61% (EE = 1.61, 95% CI: 1.09, 2.38). It is therefore possible the summary-effect estimate computed by our study might have been underestimated by these large and yet low quality studies. Pope et al. [13] also reported that their summary-effect estimate might have been underestimated by Mishra et al. [21] study.

This is the first study to review the available evidence on household solid fuel use and PTB, IUGR and miscarriage. Even though the findings reported are weakened by the small number of studies reviewed, they are consistent with the findings of previous reviews assessing the effects of ambient air pollution [8]–[12] and second-hand smoke [38], [39] on these outcomes.

### Biological Plausibility

Combustion pollutants (CO, particulate matter [PM], PAH) exert their effects on fetal growth directly by passing across the placenta or indirectly by reducing maternal lung function and increasing the risk of maternal lung disease [9], [40]. Fetuses are highly susceptible to environmental toxicants because of their differential exposure pattern and physiologic immaturity [41]. The high rate of cell proliferation and changing metabolic mechanisms during the critical phase of fetal development have been identified as the physiological process that renders the developing fetus extremely vulnerable to environmental toxicants [42].

CO reduces oxygen-carrying capacity of maternal hemoglobin, which could adversely affect oxygen delivery to fetal circulation [43]. CO crosses the placental barrier [44] and with fetal hemoglobin having greater affinity for binding CO than does adult hemoglobin [45], oxygen delivery to fetal tissues is further compromised [46]. The resultant tissue hypoxia has the potential to reduce fetal growth [43], [47]. Little is known about the mechanisms through which PM exposure influences fetal growth and development. Kannan et al. [48] have however suggested that PM exposure may cause oxidative stress, induce pulmonary and placental inflammation, alter blood coagulation factors, influence endothelial functions, and trigger hemodynamic responses which restrict fetal growth through impaired transplacental oxygen and nutrient exchange.

Regarding the effects of PAHs, Dejmek et al. [49] indicated that PAHs may directly affect early trophoblast proliferation due to their reaction with placental growth factor receptors thereby hampering feto-placental exchange of oxygen and nutrients, and consequently impairing fetal growth. Others have also hypothesized that PAHs and/or their metabolites may bind to the aryl hydrocarbon receptor resulting in antiestrogenic effects thereby disrupting the endocrine system and interfering with uterine growth during pregnancy [50], [51]. Fetal toxicity from DNA damage and resulting activation of apoptotic pathways have also been proposed [52]. According to Perera et al. [41] the finding of higher DNA adduct levels in the infant compared with the mother suggests an increased susceptibility of the developing fetus to DNA damage.

## Conclusions

Our results show that combustion of solid fuels at home increases the risk of a wide range of adverse pregnancy outcomes. Access to clean household energy solutions is the surest way to combat HAP and mitigate their adverse effects. This requires political will which is often lacking in most developing countries.

Even though we noted a high degree of consistency in study findings across the studies reviewed, major methodological limitations of most studies means further quality evidence are needed for causal inferences. All the studies reviewed applied interview methods in the ascertainment of exposure with hardly any consideration for cooking sequence and patterns, relative stability of fuel choices during pregnancy, fuel mixing/stacking, and potency of the individual fuels in causing adverse effects. The inconsistent categorization of kerosene is also a concern. These observations have consequences for the validity of the results reported by the included studies. Future research should therefore incorporate personal exposure monitoring methods and cooking activity diaries, and evaluate biomarkers of exposure. Other sources of HAP such as garbage burning at home should also be explored in future studies as well as considering the relative contribution of outdoor air pollution sources to HAP levels.

## Supporting Information

Checklist S1 **PRISMA Checklist.**
(DOC)
